# Supplementary material for: End Sequence Analysis Toolkit (ESAT) expands the extractable information from single-cell RNA-seq data
Source: Genome Res. 2016 Oct;26(10):1397–410. doi: 10.1101/gr.207902.116 (PMC5052061; doi:10.1101/gr.207902.116)
Supplement: Supplemental Material [file supp_gr.207902.116_Supp_Materials.tar.gz › ESAT-esat_v0.1/doc/html/classbroad_1_1pda_1_1annotation_1_1_g_t_f_file_parser.html]

ESAT: broad.pda.annotation.GTFFileParser Class Reference


ESAT


- Main Page
- Related Pages
- Packages
- Classes
- Files

- Class List
- Class Index
- Class Hierarchy
- Class Members

All Classes Namespaces Files Functions Variables Enumerator Pages

- broad
- pda
- annotation
- GTFFileParser

Public Member Functions |
Protected Member Functions |
List of all members

broad.pda.annotation.GTFFileParser Class Reference

Inheritance diagram for broad.pda.annotation.GTFFileParser:

[legend]

Collaboration diagram for broad.pda.annotation.GTFFileParser:

[legend]

|  |  |
| --- | --- |
| Public Member Functions | |
|  | GTFFileParser () |
|  | |
|  | GTFFileParser (String fileName) throws IOException |
|  | |
|  | GTFFileParser (String fileName, String chr) throws IOException |
|  | |
| HashMap< String, ArrayList  < String > > | getGTFGeneTranscriptMap () |
|  | |
| void | write (BufferedWriter bw, String source) throws IOException |
|  | |
| void | GTFWrite (BufferedWriter bw, String Src, boolean UseExtraFields) throws Exception |
|  | |
| HashMap< String, Locus > | getGeneIDIsoMap () |
|  | |
| Public Member Functions inherited from broad.pda.annotation.BEDFileParser | |
|  | BEDFileParser () |
|  | |
|  | BEDFileParser (String fileName) throws IOException |
|  | |
|  | BEDFileParser (String fileName, int ThreePrimeTrimSize) throws IOException |
|  | |
|  | BEDFileParser (String fileName, String chr) throws IOException |
|  | |
|  | BEDFileParser (Collection< Gene > genes) |
|  | |
|  | BEDFileParser (Map< String, Collection< Gene >> transcripts) |
|  | |
| GeneWithIsoforms | get (String geneName) |
|  | |
| List< Gene > | GetGenes () |
|  | |
| List< GeneWithIsoforms > | GetGenesWithIsoforms () |
|  | |
| IntervalTree< GeneWithIsoforms > | getChrTree (String chr) |
|  | |
| Map< String, IntervalTree  < GeneWithIsoforms > > | getIntervalTreeWithIsoforoms () |
|  | |
| Iterator< String > | getChromosomeIterator () |
|  | |
| IntervalTree< GeneWithIsoforms > | getOverlappers (Gene gene) |
|  | |
| IntervalTree< GeneWithIsoforms > | getOverlappers (LightweightGenomicAnnotation region) |
|  | |
| Iterator< GeneWithIsoforms > | getGenomicRegionOverlappers (LightweightGenomicAnnotation region) |
|  | |
| BEDFileParser | getMergedCopy () |
|  | |
| Map< String, IntervalTree  < GeneWithIsoforms > > | getMergedAnnotationMap (double minPctOverlapToMerge) |
|  | |
| Map< String, IntervalTree  < GeneWithIsoforms > > | getMergedAnnotationMap (boolean mergall) |
|  | |
| Map< String, IntervalTree  < GeneWithIsoforms > > | getAnnotationSetMap () |
|  | |
| int | getNumberOfIsoforms (String chr) |
|  | |
| int | getNumberOfIsoforms () |
|  | |
| Gene | getExactIsoform (GeneWithIsoforms loci) |
|  | |
| HashMap< String, Gene > | getNameGeneMap () |
|  | |
| BEDReader | get3PRegion (int toIn, int toOut) |
|  | |
| BEDReader | getPromoters () |
|  | |
| BEDReader | getPromoters (int toIn, int toOut) |
|  | |
| void | loadGTF (File file, String chr) throws IOException |
|  | |
| void | addRefSeq (Gene gene) |
|  | |
| void | addRefSeq (GeneWithIsoforms gene) |
|  | |
| void | addRefSeqSet (Collection<?extends Gene > collection) |
|  | |
| void | writeFullBed (String save) throws IOException |
|  | |
| void | writeFullBed (String save, boolean useExtraFields) throws IOException |
|  | |
| void | writeFullBed (BufferedWriter bw) throws IOException |
|  | |
| void | writeFullBed (BufferedWriter bw, boolean useExtraFields) throws IOException |
|  | |
| void | makeGenes () |
|  | |
| void | makeGenes (double minPctOverlapToMerge) |
|  | |
| boolean | isEmpty () |
|  | |
| boolean | contains (String geneName) |
|  | |
| boolean | containChr (String chr) |
|  | |
| void | clear () |
|  | |
| void | merge () |
|  | |
| void | collapseSingleExonTranscripts () |
|  | |
| void | IncrementScoreIfOverlap (BEDFileParser bed, int setNumber, Boolean setToZero) |
|  | |
| void | updateScrToBedScore (double score) |
|  | |
| void | filterByOverlap (Collection< Gene > genes) |
|  | |
| void | intersect (BEDFileParser bed) |
|  | |
| void | bed2gtf (BufferedWriter bw, String src) throws Exception |
|  | |
| void | bed2CuffGtf (BufferedWriter bw, String src, boolean useExtraFileds) throws Exception |
|  | |
| void | bed2gtf\_bundleOverlappers (BufferedWriter bw, String src, BEDFileParser mergedData) throws IOException |
|  | |
| void | dedup () |
|  | |
| void | minus (Collection< Gene > genes, boolean recursive) |
|  | |
| GeneWithIsoforms | remove (GeneWithIsoforms record) |
|  | |
| Map< String, Collection< Gene > > | toMap () |
|  | |
| Map< String, Collection< Gene > > | toConstituentIsoformMap () |
|  | |
| Map< String, Collection< Gene > > | toConstituentIsoformMap (boolean removeGenesWithoutConstituentIsoforms) |
|  | |
| Map< String, Collection< Gene > > | toConstituentIntroformMap () |
|  | |
| BEDFileParser | copy () |
|  | |
| BEDFileParser | overlap\_GenomicLevel (BEDFileParser other, String task, String considerOrientaion) |
|  | |
| BEDFileParser | overlapByGenomicRegion (BEDFileParser other, String considerOrientaion) |
|  | |
| BEDFileParser | mergeByGtfGeneId () |
|  | |
| BEDFileParser | equalizeTranscriptEnds (BEDFileParser other) |
|  | |
| BEDFileParser | differenceByGenomicRegion (BEDFileParser other, String considerOrientaion) |
|  | |
| BEDFileParser | removeSmallTranscripts (int minExnNum, int minTranscriptLength) |
|  | |
| BEDFileParser | removeSingeleExon () |
|  | |
| GeneWithIsoforms | getClosestUpstream (Gene annot) |
|  | |
| GeneWithIsoforms | getClosestDownstream (Gene annot) |
|  | |
| GeneWithIsoforms | getClosestUpstream (LightweightGenomicAnnotation annot) |
|  | |
| GeneWithIsoforms | getClosestDownstream (LightweightGenomicAnnotation annot) |
|  | |

|  |  |
| --- | --- |
| Protected Member Functions | |
| void | loadIsoformDataByChrToTree (File file, String chr) throws IOException |
|  | |
| void | loadIsoformDataByChrToTree (File file) throws IOException |
|  | |
| Protected Member Functions inherited from broad.pda.annotation.BEDFileParser | |
| void | loadIsoformDataByChrToTree (File file) throws IOException |
|  | |
| void | loadIsoformDataByChrToTree (File file, String chr) throws IOException |
|  | |

|  |  |
| --- | --- |
| Additional Inherited Members | |
| Static Public Member Functions inherited from broad.pda.annotation.BEDFileParser | |
| static Gene | getGeneByName (String geneName, String bedFile) throws IOException |
|  | |
| static double | getNumberOfIsoforms (IntervalTree< GeneWithIsoforms > overlapTree) |
|  | |
| static Set< Gene > | loadData (File file) throws IOException |
|  | |
| static Set< Gene > | loadData (File file, Alignments region) throws IOException |
|  | |
| static Map< String, Gene > | loadDataByName (File file) throws IOException |
|  | |
| static Map< String, Collection  < Gene > > | loadDataByChr (String file) throws IOException |
|  | |
| static Map< String, Collection  < Gene > > | loadDataByChr (File file) throws IOException |
|  | |
| static Map< String,   IntervalTree< Gene > > | loadDataByChrToTree (File file) throws IOException |
|  | |
| static Map< String, Integer > | loadChrSizes (String file) |
|  | |
| static List< String > | loadList (String file) throws IOException |
|  | |
| static List< String > | loadList (String file, boolean skipFirstLine) throws IOException |
|  | |
| static Map< Alignments, String > | loadDataLine (File file) throws IOException |
|  | |
| static Set< Annotation > | loadAlignmentData (File file) throws IOException |
|  | |
| static Set< Annotation > | loadAlignmentData (BufferedReader reader) throws IOException |
|  | |
| static Set< Annotation > | loadAlignmentData (File file, boolean ucscDownload) throws IOException |
|  | |
| static Map< String, Collection  < Annotation > > | loadAlignmentDataByChr (File file) throws IOException |
|  | |
| static Map< String,   IntervalTree< Alignments > > | loadAlignmentDataToTree (File file) throws IOException |
|  | |
| static Map< String, List  < Alignments > > | loadAlignmentDataByChrArrayList (File file) throws IOException |
|  | |
| static Map< String, Set  < Alignments > > | loadAlignmentDataByChr3Prime (File file, int extensionFactor) throws IOException |
|  | |
| static Map< String, Set  < Alignments > > | loadAlignmentDataByChr5Prime (File file, int extensionFactor) throws IOException |
|  | |
| static Map< Alignments, String > | loadDataLine (BufferedReader reader) throws IOException |
|  | |
| static void | writeBED (String save, Collection< Annotation > regions) throws IOException |
|  | |
| static void | writeBED (String save, Map< Annotation, double[]> scores) throws IOException |
|  | |
| static void | writeSortedBED (String save, Map< Annotation, double[]> scores) throws IOException |
|  | |
| static void | writeSortedBED (String save, Collection< Annotation > alignments) throws IOException |
|  | |
| static void | writeSortedBEDWithScores (String save, Map< Annotation, double[]> scores) throws IOException |
|  | |
| static void | writeBEDWithScores (String save, Map< Annotation, double[]> scores) throws IOException |
|  | |
| static void | writeBED (String save, Collection< Annotation > alignments, boolean sort) throws IOException |
|  | |
| static void | writeBED (String save, Map< Annotation, double[]> scores, boolean withScores, boolean sort) throws IOException |
|  | |
| static void | writeFullBED (String save, Collection< Gene > genes) throws IOException |
|  | |
| static void | writeFullBED (String save, Map< Gene,?extends Object > genes) throws IOException |
|  | |
| static Map< String,   IntervalTree< Gene > > | makeIntervalTreeFor3Prime (File geneFile, int size) throws IOException |
|  | |
| static boolean | isOverlapCompatible (Gene first, Gene second, double minPctOverlap) |
|  | |
| static void | main (String[] args) throws IOException |
|  | |
| static Map< String, BEDFileParser > | uploadSetOfFiles (String bedLst) throws IOException |
|  | |
| static BEDFileParser | expanedUtrs (BEDFileParser bed, Integer utr1, Integer utr2) |
|  | |
| static BEDFileParser | readSiphyOutToBed (String siphyFile) throws NumberFormatException, IOException |
|  | |
| static Gene | getGene (String geneFile, String geneName) throws IOException |
|  | |

## Constructor & Destructor Documentation

|  |  |  |  |  |
| --- | --- | --- | --- | --- |
| broad.pda.annotation.GTFFileParser.GTFFileParser | ( |  | ) |  |

|  |  |  |  |  |  |
| --- | --- | --- | --- | --- | --- |
| broad.pda.annotation.GTFFileParser.GTFFileParser | ( | String | *fileName* | ) | throws IOException |

Here is the call graph for this function:

|  |  |  |  |
| --- | --- | --- | --- |
| broad.pda.annotation.GTFFileParser.GTFFileParser | ( | String | *fileName*, |
|  |  | String | *chr* |
|  | ) |  | throws IOException |

Here is the call graph for this function:

## Member Function Documentation

|  |  |  |  |  |
| --- | --- | --- | --- | --- |
| HashMap<String,Locus> broad.pda.annotation.GTFFileParser.getGeneIDIsoMap | ( |  | ) |  |

Here is the call graph for this function:

|  |  |  |  |  |
| --- | --- | --- | --- | --- |
| HashMap<String, ArrayList<String> > broad.pda.annotation.GTFFileParser.getGTFGeneTranscriptMap | ( |  | ) |  |

Here is the call graph for this function:

|  |  |  |  |
| --- | --- | --- | --- |
| void broad.pda.annotation.GTFFileParser.GTFWrite | ( | BufferedWriter | *bw*, |
|  |  | String | *Src*, |
|  |  | boolean | *UseExtraFields* |
|  | ) |  | throws Exception |

Write a GTF using only the attributes in extraFields (not modifying the gene\_id, transcript\_id as GTF2BED does)

Parameters
:   |  |  |
    | --- | --- |
    | bw | BufferedWriter |
    | Src | string to annotate src field |
    | UseExtraFields | - if false call gtf2bed ; if true assumes has gene id and transcript id as maybe others |

Exceptions
:   |  |  |
    | --- | --- |
    | Exception |  |

Here is the call graph for this function:

|  |  |  |  |
| --- | --- | --- | --- |
| void broad.pda.annotation.GTFFileParser.loadIsoformDataByChrToTree | ( | File | *file*, |
|  |  | String | *chr* |
|  | ) |  | throws IOException |

protected

Here is the call graph for this function:

Here is the caller graph for this function:

|  |  |  |  |  |  |
| --- | --- | --- | --- | --- | --- |
| void broad.pda.annotation.GTFFileParser.loadIsoformDataByChrToTree | ( | File | *file* | ) | throws IOException |

protected

Here is the call graph for this function:

|  |  |  |  |
| --- | --- | --- | --- |
| void broad.pda.annotation.GTFFileParser.write | ( | BufferedWriter | *bw*, |
|  |  | String | *source* |
|  | ) |  | throws IOException |

Here is the call graph for this function:

---

The documentation for this class was generated from the following file:

- java/broad/pda/annotation/GTFFileParser.java


---

Generated on Fri Aug 15 2014 15:10:58 for ESAT by  

 1.8.7
